# The Speed of Optic Flow Stimuli Influences Body Sway

**DOI:** 10.3390/ijerph191710796

**Published:** 2022-08-30

**Authors:** Milena Raffi, Aurelio Trofè, Andrea Meoni, Alessandro Piras

**Affiliations:** 1 Department of Biomedical and Neuromotor Sciences, University of Bologna, 40126 Bologna, Italy; 2 Department of Quality of Life, University of Bologna, 47921 Rimini, Italy

**Keywords:** posture, visual perception, visual processing, postural control, heading perception, electromyography, stabilometry, body oscillation, gender differences, visual system

## Abstract

Optic flow is a perceptual cue processed for self-motion control. The aim of this study was to investigate whether postural control is modulated by the speed of radial optic flow stimuli. The experiments were performed on 20 healthy volunteers using stabilometry and surface electromyography (EMG). The subjects were instructed to fixate a central fixation point while radial optic flow stimuli were presented full field, in the foveal and in the peripheral visual field at different dots speed (8, 11, 14, 17 and 20°/s). Fixation in the dark was used as control stimulus. The EMG analysis showed that male and female subjects reacted to the stimuli with different muscle activity (main effects for gender, muscle and laterality: *p* < 0.001). The analysis of the center of pressure (COP) parameters showed that optic flow stimuli had a different effect on the left and right limbs of males and females (main effects of laterality: *p* < 0.015; interaction effects of gender and laterality: *p* < 0.016). The low speed of optic flow stimuli (8 and 11°/s) evoked non-uniform directions of oscillations especially in peripheral stimulation in all subjects, meaning that optic flow simulating slow self-motion stabilizes body sway.

## 1. Introduction

When we move in the environment, the retina undergoes a whole-field stimulation, the optic flow, which depends on the speed and direction of our movement and on the structure of the visual scene [1]. Neurophysiological studies performed in the past decades showed that several areas of the monkey and human brain possess optic flow neurons activated by different modalities [2,3,4,5,6,7,8,9,10]. Thus, it is now well documented that the perception of self-motion is multimodal, involving more than simply the combination of motor action and visual feedback [11].

Postural control requires the interaction of many sensory modalities, with the most important role of visual input [12,13]. The integration of such signals generates the typical body oscillation known as body sway. The body sway is regulated by the neuromotor system with the important role of creating coordinated muscle activity to control posture. A well-stabilized posture is necessary to provide support for voluntary limb, head, or trunk movements. Postural control and balance involve the control of the body’s position in space for stability and orientation.

The optic flow field provides information about the speed, direction and distance of self-motion [14,15]. A few studies aimed at investigating the effect of simulated self-motion by an optic flow stimulus showed that optic flow influences the amount of induced postural sway [16,17,18]. The speed of the optic flow is an important perceptual input for determining heading direction and avoiding obstacles during self-motion. In our previous study, we used optic flow stimuli with dissimilar speed gradients to simulate rightward and leftward self-motion, showing that such stimuli influence body sway [19]. Other studies showed that the speed gradient of an optic flow pattern influences the magnitude of postural sway [17,20,21]. A recent paper by Engel et al. [22] supports the claim of simultaneously co-existing modes of body sway depending on the frequency of visual perturbation. The authors suggest that the human body behaves like a single-link inverted pendulum at low motion frequencies, whereas optimal adaptation is achieved by multi-link coordination of body segments towards higher frequencies.

An observer usually moves in the environment following defined paths [23]. Such paths provide the necessary information for generating successful self-motion trajectories via feedback visual mechanisms. It has been shown that during walking, human subjects attempt to optically equalize the differences in the optic flow vector speeds between the left and right visual fields [24,25,26,27]. Optic flow speed from a ground plane has an important effect on the trajectories that human subjects take when steering [28]. The ability of steering is also influenced as it was found to systematically vary according to optic flow speed [29]. A few laboratory experiments performed on human subjects walking on a treadmill showed that changes in gait speed occur when optic flow speed is altered. For example, walking speed slows down when optic flow velocity increases [30,31].

The rationale of the present study arises from the knowledge that the speed information from the optic flow field can vary considerably across different environments and daily situations. Pickhinke et al. [32] showed that the manipulation of optical flow speed has an effect on postural control during locomotion, meaning that if self-motion perception becomes less predictable, postural control during locomotion becomes more variable. We also know that different retinal regions have a different effect on body balance control; although the literature is a bit controversial, peripheral visual stimuli seem to better stabilize posture (cfr. [33] for review). In the present study, we examined the potential effect of speed on the relationship between optical flow and postural control during quiet standing. Given that it is well known that males and females react to the optic flow with a different muscular activation and COP values [19,34,35], we also verified the effect of optic flow speed on gender. We hypothesized that postural stability during quiet stance would be modified by the different speeds of optic flow stimuli. To assess this hypothesis, our subjects viewed radial expanding optic flows at different speeds (8, 11, 14, 17 and 20°/s) presented full field, in the central and in the peripheral visual field.

## 2. Materials and Methods

For this study, we recruited 20 healthy volunteers (10 females and 10 males) who did not receive any compensation. The participants were the same as in the previous study [36], with the addition of another subject. None of them were taking medications or supplements. None of the subjects reported physical deficit or muscular injury at the time of the study. The participants’ age ranged from 21 to 35 years (average 27.9), and average height and weight including standard deviation were 171 ± 7 cm and 64.7 ± 9.89 kg, respectively. The average BMI of the subjects was 21.91 ± 2.30. All subjects had normal or corrected to normal vision. The hand and foot laterality of each subject was assessed by a laterality questionnaire before the beginning of the experiment. We used a revised version of the standardized Waterloo Footedness Questionnaire (WFQ) and Waterloo Handedness Questionnaire (WHQ) [37,38] with the following formula:

[(right preference − left preference)/(right preference + left preference)] × 100

A positive laterality index was indicative of a right dominance, while a negative index was indicative of a left dominance.

Written informed consent to participate in the study was signed before the beginning of recordings. The experimental protocol was approved by the Institutional Ethic Committee of the University of Bologna. The experiments were performed in accordance with the ethical standards laid down in the 1964 Declaration of Helsinki.

### 2.1. Optic Flow Stimuli

**Figure 1:**
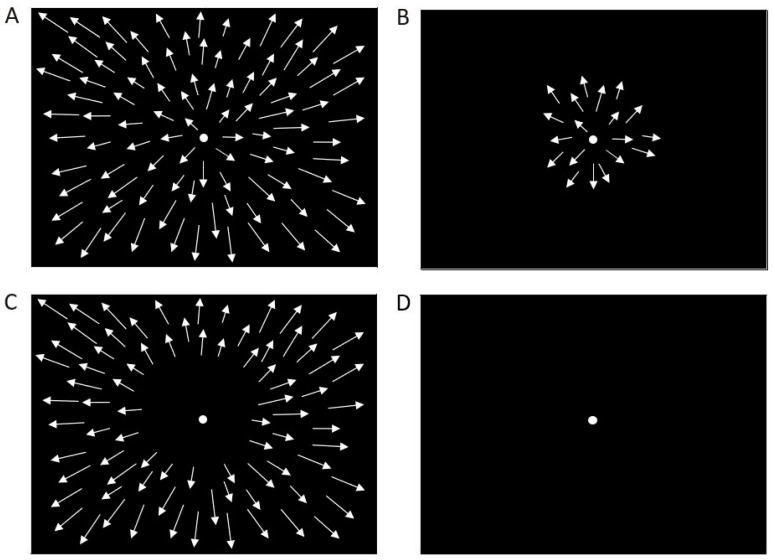
Radial optic flow and control stimuli. (**A**) Full-field stimulus. (**B**) Foveal stimulus. (**C**) Peripheral stimulus. (**D**) Baseline (control). Full, foveal and peripheral stimuli were presented at different speeds: 8, 11, 14, 17 and 20°/s. The arrows represent the velocity vectors of moving dots. The dots were moving radially from the center to the periphery following a line on each of the 360°.

The stimuli used in this study were identical to those used in a previous study [36] in which we analyzed the effects of optic flow speed on microsaccades using the EyeLinkII eye tracking system (SR Research Ltd., Mississauga, ON, Canada). Briefly, radial optic flow visual stimuli consisted of white dots (1.3 cd/m^2^, size 0.4°) presented full field, in foveal or in peripheral visual field (Figure 1A–C). The stimuli were retro-projected on a translucent screen that covered 135 × 107° of the visual field. The subjects were instructed to look at a fixation point projected on the screen (Figure 1). The fixation point position was adjusted according to the height of each subject. The experiments were performed in a dark room. To study the influence of different optic flow speeds, we varied the dot speed in all three stimuli obtaining 15 different conditions: the tested speed of the optic flow stimulus was 8, 11, 14, 17 and 20°/s. These optic flow stimuli always contained a speed gradient, with the speed increasing from the center to the periphery.

We used simple fixation on a dark screen as a control stimulus (Figure 1D). The optic flow stimuli were made using Matlab psychophysical toolbox (The Mathworks Inc., Natick, MA, USA). We recorded 2 repetition for both baseline and optic flow stimuli with different speeds; thus, each subject performed 32 trials. Each trial lasted 30 s. The stimuli were randomly presented to prevent selection bias. The randomization was identical for all subjects to create a homogeneous treatment between participants, without involving any potential biases or judgments.

### 2.2. Recordings

Experiments were performed in a quiet room with stable temperature (21 °C; 52% of humidity). The subjects were asked to avoid drinking caffeinated beverages before the experimental procedures and were instructed to avoid strenuous activity and alcohol in the 12 h preceding the test.

Before recordings, the subjects were instructed to fixate on the fixation point and not to resist to the optic flow stimulus.

The participants were placed in a standing posture on two Kistler force platforms in front of the translucent screen in which the optic flow visual stimuli were back-projected. On the top of the platforms, we identified a line in which they had to place the upper extremity of their halluces. All subjects placed their feet in the same position.

Electromyography data were recorded using disposable Ag/AgCl electrodes, 32 × 32 mm used in a bipolar configuration. The electrodes were positioned on the muscular belly of the following muscles: right paraspinal-C4 (RC4), left paraspinal-C4 (LC4), right trapezius descendens (RTD), left trapezius descendens (LTD), right tibialis anterior (RTA), left tibialis anterior (LTA), right soleus (RSOL), and left soleus (LSOL). EMG data were acquired at 1000 Hz by FREE1000 EMG (BTS Bioengineering Inc.). We acquired the maximum voluntary contraction (MVC) of each muscle using isometric machines. The peak of the MCV was used for the normalization of EMG activity.

Stabilometric data were acquired at a sampling rate of 1000 Hz using two Kistler force platforms (Kistler Instrument, Winterthur, Switzerland). During the recording, the subjects stood with a foot on each platform.

### 2.3. Data Analysis

The EMG signals were positively rectified and band-pass-filtered (Butterworth, 20–450 Hz) using SMART Analyzer (BTS Bioengineering Inc., Garbagnate Milanese, Italy). Each trial was normalized to the peak of the MVC. The normalized root mean square (RMS) values were calculated in 100 ms bins from the EMG signals using Matlab (The Mathworks Inc., Natick, MA, USA). A repeated measures ANOVA was performed on the normalized EMG signals: muscle (RC4, LC4, RTD, LTD, RTA, LTA, RSOL, LSOL) and side (left, right) were set as within factors, while stimuli (full-field, foveal, peripheral), speed (8, 11, 14, 17, 20°/s) and gender (male, female) were set as between factors. Effect sizes were calculated using partial eta squared (η_p_^2^), and means were considered significantly different at *p* < 0.05.

Stabilometric data were low-pass-filtered at 15 Hz. We analyzed the antero-posterior (AP) oscillation, the medio-lateral (ML) oscillation, the COP area and the COP speed. We first computed the values of the four COP parameters in each subject for each stimulus. Then, we averaged the values of each COP parameter for all subjects in each stimulus. A repeated measures ANOVA was performed on each COP parameter separately (AP, ML, COP area and COP speed): the COP parameter was set as within factors, while stimuli (full-field, foveal, peripheral), speed (8, 11, 14, 17, 20°/s) and gender (male, female) were set as between factors. Effect sizes were calculated using partial eta squared (η_p_^2^), and means were considered significantly different at *p* < 0.05.

To assess the effect of optic flow speed on body sway, we computed the maximal variance direction, which corresponds to the prevalent direction of oscillation, according to the following formula (Chiari et al., 2007):

Max variance direction = atan(V_ML_/V_AP_)     {+π if V_ML_/V_AP_ < 0}

where V_ML_ and V_AP_ are the eigenvectors corresponding to the maximum eigenvalues of

C = covariance (ML[n], AP[n]), where n is the sample index.

Data of the prevalent direction of oscillation were then analyzed by circular statistics, where 0° corresponded to rightward oscillation, 90° to anterior oscillation, 180° to leftward oscillation and 270° to posterior oscillation (ORIANA, Kovach Computing Services). The consistency of the mean vectors distribution was assessed with the Rayleigh test of uniformity, and results were considered significant at *p* < 0.05.

## 3. Results

The analysis of the laterality questionnaires showed that 17 participants were strongly right-handed and right-footed with values above 78. Three subjects scored −44, −55 and −89, respectively, indicating a strong left laterality for only one subject.

### 3.1. EMG Signals

The results of the repeated measures ANOVA (see Methods) revealed significant main effects for muscle (F_3,864_ = 570.09; *p* < 0.001; η_p_^2^ = 0.664), body side (F_1,288_ = 12.48; *p* < 0.001; η_p_^2^ = 0.042) and gender (F_1,288_ = 14.649; *p* < 0.001; η_p_^2^ = 0.048). Significant interaction effects were observed for muscle × body side (F_3,864_ = 23.08; *p* < 0.001; η_p_^2^ = 0.074), muscle × gender (F_3,864_ = 19.25; *p* < 0.001; η_p_^2^ = 0.063) and muscle × body side × gender (F_3,864_ = 6.472; *p* < 0.001; η_p_^2^ = 0.022). No significant effect was observed for stimulus.

Regarding the interactions, the post hoc verification showed the following significant effects for muscle (paired sample *t*-test): RSOL vs. LSOL, *t*(31) = 1.69, *p* = 0.039; RTA vs. LTA, *t*(31) = 1.69, *p* = 0.002; all other comparisons resulted significant at *p* < 0.001. The post hoc verification showed the following significant effects for gender (independent sample *t*-test: female vs. male): *t*(30) = 1.69, RC4, *p* < 0.001; *t*(30) = 1.69, LC4, *p* < 0.001; *t*(30) = 1.69, RTD, *p* < 0.001; *t*(30) = 1.69, LTD, *p* < 0.001; *t*(30) = 1.69, RTA, *p* < 0.001; *t*(30) = 1.69, LTA, *p* < 0.001; *t*(30) = 1.69, RSOL, *p* < 0.001; *t*(30) = 1.69, LSOL, *p* < 0.001. The post hoc verification showed the following significant effects for side (independent sample *t*-test: left vs. right): female C4, *t*(30) = 1.69, *p* < 0.001; male C4, *t*(30) = 1.69, *p* < 0.001; female TD, *t*(30) = 1.69, *p* < 0.001; male TD, *t*(30) = 1.69, *p* = 0.041; female TA, *t*(30) = 1.69, *p* < 0.001; male TA, *t*(30) = 1.69, *p* = 0.005; male SOL, *t*(30) = 1.69, *p* = 0.003. The above-described statistics indicate that the muscles activity was different in males and females in both body sides.

**Figure 2:**
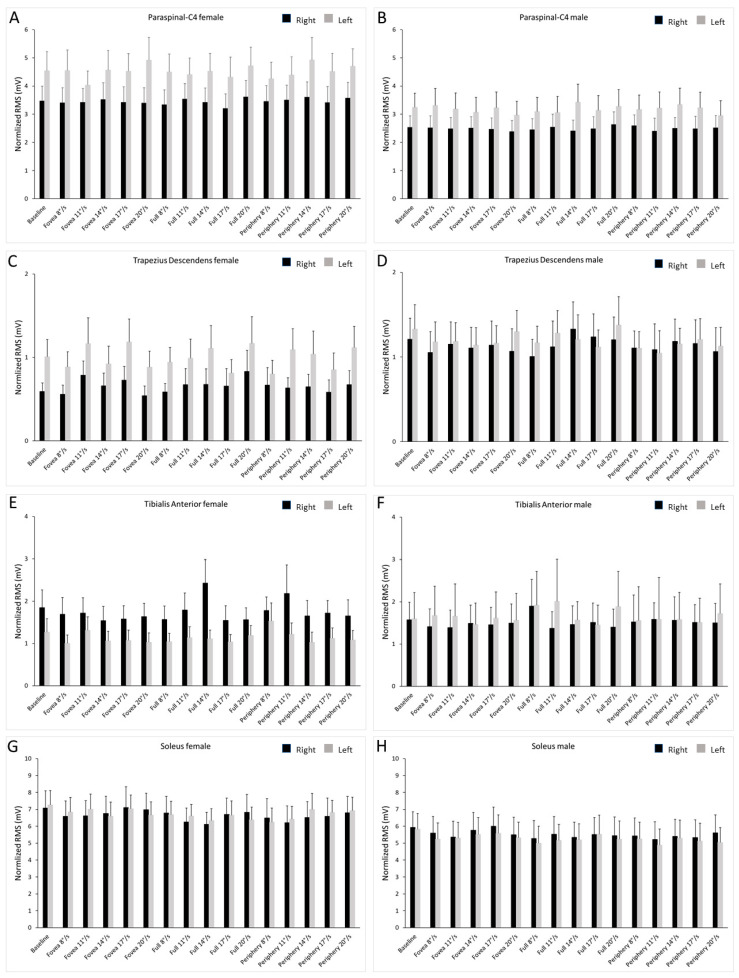
Bar graph of normalized EMG traces in the left and right limb of male and female subjects. Values are shown during optic flow stimuli at different speeds and baseline. The black bars represent the right muscles, and the gray bars represent the left muscles. Data are shown as mean ± SE. (**A**) Activity of the paraspianal-C4 in females. (**B**) Activity of the paraspianal-C4 in males. (**C**) Activity of the trapezius descendens in females. (**D**) Activity of the trapezius descendens in males. (**E**) Activity of the tibialis anterior in females. (**F**) Activity of the tibialis anterior in males. (**G**) Activity of the soleus in females. (**H**) Activity of the soleus in males.

Figure 2 shows the average values of the normalized RMS. The female participants showed a higher activity of the LC4, and the males of the LTD. The females also presented a higher activity of the RTA with respect to LTA.

### 3.2. COP Parameters

**Figure 3:**
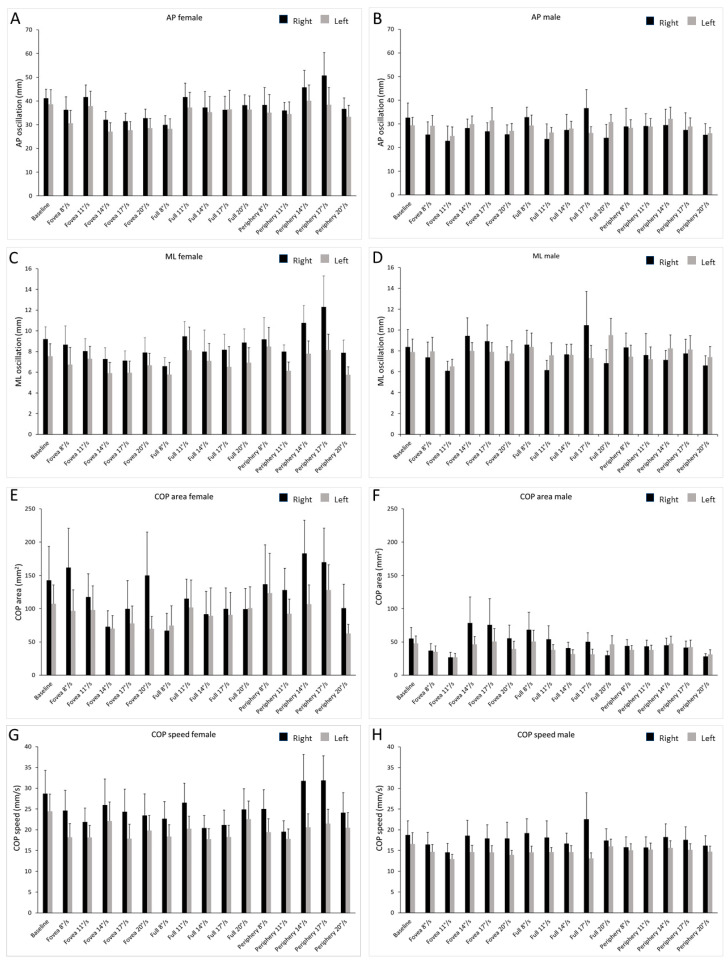
Bar graph of COP parameters in the left and right limb of male and female subjects. Values are shown during optic flow stimuli at different speeds and baseline. The black bars represent the right limb, and the gray bars represent the left limb. Data are shown as mean ± SE. (**A**) AP oscillations in females. (**B**) AP oscillations in males. (**C**) ML oscillations in females. (**D**) ML oscillations in males. (**E**) COP area in females. (**F**) COP area in males. (**G**) COP speed in females. (**H**) COP speed in males.

The AP oscillation (Figure 3A,B) revealed a significant main effect of gender (F_1,288_ = 21.218; *p* < 0.001; η_p_^2^ = 0.069) with a greater value of the female participants (Female = 35.56 ± 1.09 vs. Male = 27.93 ± 1.09). The results also showed an interaction between body side and gender (F_1,288_ = 7.173; *p* = 0.008; η_p_^2^ = 0.024). The post hoc verification showed a significant effect of gender (paired sample *t*-test, *t*(19) = 1.72, *p* = 0.03), meaning that the AP oscillation was greater in females than in males but only on the right side.

The ML oscillation (Figure 3C,D) showed a significant main effect of body side (F_1,288_ = 6.036; *p* = 0.015; η_p_^2^ = 0.021), with a greater value of the right side (Right = 8.537 ± 0.478 vs. Left = 7.35 ± 0.232). We also found an interaction between body side and gender (F_1,288_ = 5.843; *p* = 0.016; η_p_^2^ = 0.020), in which the post hoc verification showed a significant effect of side (paired sample *t*-test, *t*(19) = 1.72, *p* = 0.01), meaning that the ML oscillation was greater in the right side only for females.

The COP area (Figure 3E,F) showed significant main effect of gender (F_1,288_ = 7.874; *p* = 0.005; η_p_^2^ = 0.027), validating the greater value of the female participants (Female = 151.76 ± 25.14 vs. Male = 49.23 ± 25.23).

The COP speed (Figure 3G,H) showed a significant main effect for body side (F_1,288_ = 16.86; *p* < 0.001; η_p_^2^ = 0.055) and gender (F_1,288_ = 24.448; *p* < 0.001; η_p_^2^ = 0.078), with greater values for right side of body (Right = 21.83 ± 1.05 vs. Left = 17.31 ± 0.471) and female participants (Female = 22.95 ± 0.91 vs. Male = 16.19 ± 0.91).

### 3.3. Prevalent Direction of Oscillation

**Figure 4:**
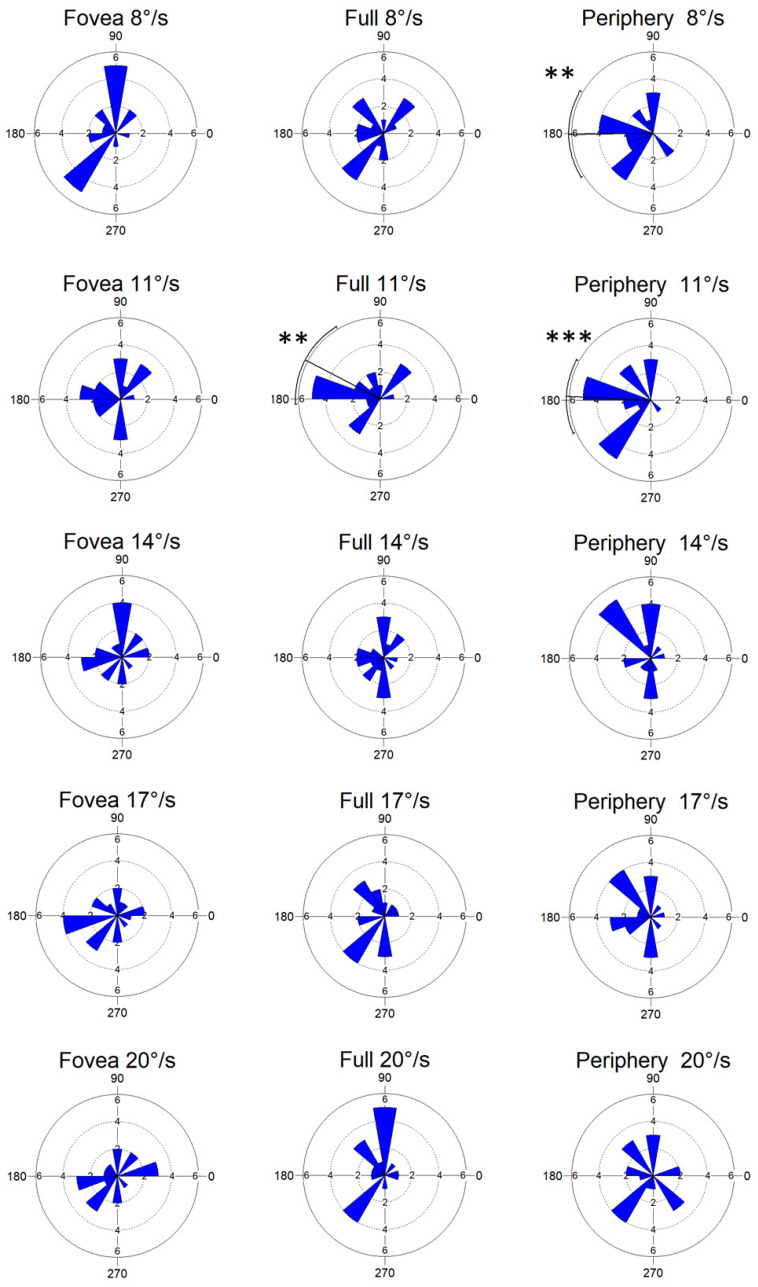
Distributions of preferred directions of oscillation for optic flow stimuli at different speeds. Rose diagrams show the frequency distribution of the mean vectors of all trials computed for each stimulus in each subject. The diagram plots lines on each of the 360° of a compass distribution, with the length proportional to the number of values in that direction. The solid line crossing each diagram indicates the mean vector when significant, whereas the curved line outside the circle indicates circular SD. The bars are 20° wide. Asterisks indicate significant values with non-uniform distribution (Rayleigh test of uniformity).

The distribution of the prevalent directions of oscillation during the optic flow stimulation at different speeds is shown in Figure 4. Three stimuli evoked non-uniform directions of oscillation according to the Rayleigh test of uniformity: full-field optic flow at a speed of 11°/s (*p* = 0.003), and peripheral optic flow at 8°/s (*p* = 0.003) and 11°/s (*p* < 0.001).

## 4. Discussion

The aim of this study was to assess if the manipulation of optic flow speed affects postural control during quiet standing. Our results showed that lower speed of optic flow stimuli (8 and 11°/s) evokes non-uniform directions of oscillations, especially during peripheral stimulation. Further, the male and female subjects reacted to the optic flow with different postural asset and muscular activation.

### 4.1. Optic Flow Speed Effect on the Direction of Oscillation

The present data showed a strong effect of optic flow speed on the direction of postural sway. The analysis revealed that lower speed of optic flow stimuli (8 and 11°/s) evokes non-uniform directions of oscillations, mostly in peripheral stimulation (Figure 4). High speed of optic flow stimuli (>14°/s) evoked uniform directions of oscillation, likely indicating that high optic flow speed has a destabilizing effect on postural control. As described in Methods, we analyzed the maximal variance direction in the entire postural trace (30 s), so the finding of the stabilizing (8 and 11°/s) and destabilizing (>14°/s) effect of speed is indicative of the activation of specific neuronal pathways generating similar, in case of low-speed, or dissimilar, in case of high-speed, oscillations.

As mentioned in the introduction, the retinal regions have different effects on body balance control. These functional differences arise from the anatomical organization of the retina. The relative densities of the types of ganglion cells vary with retinal eccentricity and peripheral ganglion cells project to the magnocellular layers of the lateral geniculate nucleus originating the dorsal stream [39]. This visual pathway involves occipito-parietal neurons, which process information related to self-motion perception, depth and spatial orientation [40]. The anatomo-physiological organization of the visual system supports the view that peripheral stimuli are more effective in stabilizing posture due to the involvement of the parietal cortex circuits (crf. [33] for review).

### 4.2. Muscle Activity and COP Parameters

Our hypothesis was that the speed of optic flow stimuli would modulate the postural control. Although we found the effect of speed in the analysis of the prevalent direction of oscillation, we did not find any effect of speed or optic flow stimuli on muscle activity or COP parameters. In the past years, in our laboratory, we performed quite a few experiments aimed at uncovering the optic flow effects on postural control and we found that different optic flow stimuli evoke different postural oscillations, but, at the same time, we never found a modulation of optic flow stimuli on postural muscles or COP parameters. This leads to few considerations.

Firstly, the postural asset. Our results seem to indicate that postural asset is well established during neural development, indicating that a person has his/her own motor coordination dynamics to control posture [19]. This view agrees with that of previous studies. Stamenkovic and Stapley [41] indicated that the nervous system is aware of the dynamics of the task before motor task execution, and thus postural adjustments of non-specific body segments may not be necessary for countering the reactive force. Further, studying the development of the neural system, Gilmore et al. [42] investigated optic flow processing in children; the authors suggested that the brain segregates the processing of optic flow pattern from speed and that an adult-like pattern of neural responses to optic flow emerges by early to middle childhood. Fesi et al. [43] evaluated the effects of optic flow speed (2–16°/s) on visually evoked potential responses in adults, showing that radial optic flow evokes strong neural responses, and the location of activity varies by speed.

Secondly, the learning effect. Our experimental protocol applied a blocked design where the subject experienced the same type of stimulation repetitively. Dionne and Henriques [44] showed that, over a sequence of trials, human subjects progressively learn to correct visual perturbations. Perturbations to ongoing movement showed learning-dependent compensatory responses (cfr. [45] for review). All these findings indicate that human subjects may learn to generate a response that mirrors the perturbation and enables them to move as if they were not perturbed [46], so it is possible to hypothesize that our subjects learned to respond to the optic flow over time.

Lastly, the trial duration. In our study protocol each trial lasted 30 s, which is the well-established paradigm for studying postural control. However, as discussed above, the neural system tends to compensate external perturbations; thus, mean values computed over the entire trial duration may not be adequate to uncover potential phasic motor responses. Another indication that a lack of stimulus speed effect on COP parameters and muscle activity could be due to the analysis of the entire trial duration arises from the scientific literature. Several studies showed that different speed of optic flow stimuli alter postural sway [17,21]. Holten et al. [20] performed an experiment in which their subjects viewed optic flow stimuli containing different speed gradients between center and peripheral flow field presented at different speeds. The authors showed that decreasing the speed gradient of the optic flow stimulus produces a higher body sway and that higher speed of optic flow stimuli evokes more postural sway. However, in contrast to the present study, the trial duration in the study of Holten et al. was 4 s, allowing the analysis of the perturbation evoked by the optic flow without the influence of adaptation mechanisms. Indeed, previous findings indicated a sense of self-motion (vection) latency from 4 to 7 s after the onset of moving visual stimuli [47,48].

### 4.3. Gender Differences on Postural Control

We decided to test neck, back and leg muscles to verify the involvement of the head and the upper body on the COP parameters and postural sway. Our results are in agreement with those of previous studies [49,50] that observed significant differences in neck posture related to gender. Those findings allowed the authors to suggest that male and female subjects vary in how they adopt flexed neck postures. Reddy et al. [51] performed an experiment on neck strength testing using MR imaging. The results showed that males were about 65% stronger and had significantly larger muscles. These data likely explain why our male subjects had a lower paraspinal-C4 activity with respect to females, given that a larger muscle needs a minor activity for the same required strength. Reddy et al. also suggested that males and females exhibit distinct size-strength relationships, highlighting the need for sex-specific models and analyses. It has to be noted however, that our results differ from those of a previous study regarding the activity of the trapezius. We found that female subjects have a lower activity with respect to males, while Cui et al. showed opposite results [52]. This difference could be attributed to the experimental protocol; indeed, the subjects of Cui et al. were required to complete a 90 min text typing task while standing. It is possible that standing for so long time requires motor adaptation mechanisms not visible in a 30 s task.

## 5. Conclusions

In this study, we showed that low speed of optic flow stimuli has an effect on postural oscillations, especially in peripheral stimulation. These data confirm that peripheral retina plays a critical role in self-motion perception. The lack of significant speed effects on postural muscles could be due to motor adaptation mechanisms occurring during quite stance. It is thus possible to hypothesize that such mechanisms are used in heading perception. All things considered, the present results open new questions about the postural strategies used to respond to optic flow. A detailed analysis in the time domain is required to deepen the knowledge on postural control mechanisms.

## Data Availability

Not applicable.
